# Pre-Procedure Thrombocytopenia and Leukopenia Association with Risk for Infection in Image-Guided Tunneled Central Venous Catheter Placement

**DOI:** 10.3390/tomography8020052

**Published:** 2022-03-01

**Authors:** Abigail Luman, Keith B. Quencer, Claire Kaufman

**Affiliations:** 1 School of Medicine, University of Utah, Salt Lake City, UT 84132, USA; abigail.luman@hsc.utah.edu; 2 Department of Radiology and Imaging Sciences, School of Medicine, University of Utah, Salt Lake City, UT 84132, USA; keith.quencer@hsc.utah.edu

**Keywords:** central venous catheter, leukopenia, thrombocytopenia, infection, interventional radiology

## Abstract

Placement of image-guided tunneled and non-tunneled large-bore central venous catheters (CVCs) are common procedures in interventional radiology. Although leukopenia and/or thrombocytopenia are common at the time of placement, the roles these factors may have in subsequent catheter-related infection have yet to be investigated. A single-institution retrospective review was performed in patients who underwent CVC placement in interventional radiology between 11/2018–6/2019. The electronic medical record was used to obtain demographics, procedure details, pre-placement laboratory values, and the subsequent 90-day follow-up. A total of 178 tunneled and non-tunneled CVCs met inclusion criteria during this time period. White blood cell (WBC) and platelet counts were found to be significant risk factors for subsequent infection. Administration of pre-procedure antibiotics was not found to be a significant factor for subsequent infection (*p* = 0.075). Leukopenia and thrombocytopenia at the time of CVC placement are both risk factors of line infection for tunneled large-bore CVCs. This should lead to the consideration of using a non-tunneled CVC when clinically feasible, or the delayed placement of these catheters until counts recover.

## 1. Introduction

Central line-associated bloodstream infections (CLABSIs), previously found to be the third leading cause of hospital-acquired infections, are associated with high morbidity and mortality [1,2]. Image-guided, large-bore central venous catheters (CVCs) can be placed directly, via the internal jugular, subclavian or femoral veins (non-tunneled), or tunneled through the subcutaneous tissues before entry into a central vein (tunneled central venous catheters). These CVCs are commonly placed by interventional radiologists, not only for hemodialysis or plasmapheresis, but also in the setting of hematologic malignancy, such as for hematopoietic cell transplantation in which preparative myeloablative conditioning results in long-lasting pancytopenia [3]. Additionally, patients with end-stage renal disease (ESRD) have immune dysfunction that is associated with a higher risk of morbidity and mortality from infections [4].

There is conflicting data regarding an association of neutropenia and thrombocytopenia during the placement of an implantable CVC, specifically ports, and the subsequent risk of CLABSI in this population is controversial. A study performed in 2007 involving 195 pediatric patients showed a significantly higher rate of early CVC or central venous port removal secondary to CLABSI or other complications in neutropenic patients [5]. A more recent study of 183 pediatric patients did not find an increased risk of early removal of central venous ports in a neutropenic patient [6]. However, Bamba et al. and Skummer et al. both found that leukopenia at the time of port placement was a risk factor for subsequent early port infection [7,8].

The placement of a tunneled CVC is categorized as a low-risk procedure for bleeding, and can be performed in patients with thrombocytopenia although platelet transfusion is recommended if <20 × 10^9^ L [9,10,11]. Major bleeding events associated with this procedure are indeed rare, and a safe threshold of thrombocytopenia is unclear [12]. Additionally, the practice of prophylactically administering platelets prior to CVC placement is widely varied, based on clinician decision-making [13]. This has led to a call for additional research investigating risk factors, specifically thrombocytopenia, for bleeding in the placement of tunneled CVCs.

We hypothesized that, similar to port placement, there would be higher rates of CLABSI in patients with leukopenia and/or thrombocytopenia. This single-institution retrospective review investigates the rates of complications including CLABSI, bleeding and mortality, in neutropenic and/or thrombocytopenic patients undergoing tunneled and non-tunneled CVC placement.

## 2. Materials and Methods

### 2.1. Patients

This single-institution retrospective study was approved by the institutional review board (IRB) at our institution and carried out in compliance with the Health Insurance Portability and Accountability Act (HIPAA) guidelines. Patients who underwent central venous catheter placement in interventional radiology at our tertiary care hospital between November 2018 and June 2019 were identified using CPT codes. Patients were excluded if their catheter was placed in a location other than the interventional radiology angiography suite or by another service, if the catheter was an exchange and not primary placement, if they were younger than 18 years old, had a small-bore catheter placed (defined as <9 French), or if there were no labs within 30 days of placement.

### 2.2. Procedure

All catheters were placed under ultrasound and fluoroscopic guidance in the interventional radiology suite, by or under the supervision of a fellowship-trained interventional radiologist or a dedicated interventional radiology physician assistant. All patients were prepped and draped in the usual sterile fashion using chlorohexidine or betadine to clean the skin. Disposable materials were used for all procedures. Venous access was obtained using continuous ultrasound guidance and a 21-gauge micropuncture needle, most commonly via the right or left internal jugular vein. The remainder of the procedure was performed with fluoroscopic guidance. Not all patients were given periprocedural antibiotics, although this was documented. Procedures were performed with local lidocaine and/or moderate sedations utilizing fentanyl and midazolam, depending on clinical scenario, patient desire, and type of catheter placed.

### 2.3. Data Collection and Statistical Analysis

The electronic medical record was used to obtain patient demographics, procedure details (type of catheter placed, location of insertion, indication, and periprocedural antibiotics), laboratory values at the time of catheter placement, and subsequent 90-day follow-up. Documented positive blood cultures, bleeding events and mortality within the 90 day follow up were recorded. Patients were categorized as those with known infection for other reasons, patients with no sign of infection, patients with positive blood cultures after placement, and patients with fever of unknown origin. Bleeding events were distinguished in severity with the following criteria. Mild bleeding was mentioned in documentation and required no intervention other than dressing changes. Moderate bleeding events required non-invasive intervention, such as the application of a hemostatic wafer. Severe bleeding events required invasive intervention, removal of the CVC, or transfusion of blood products. Statistical analysis was performed using a two-tailed *t*-test to evaluate the significance of the lab values in those with positive blood cultures and those without evidence of infection. Significance was defined as a *p* value of ≤0.05. Given the small sample size, a Fischer exact test was used to evaluate significance (*p* ≤ 0.05) of antibiotic administration, or lack thereof, in patients with positive blood cultures.

## 3. Results

A total of 400 catheters were reviewed, from 12 November 2018 to 28 June 2019. Of these: 178 catheters met inclusion criteria, 97 tunneled CVCs, and 81 non-tunneled CVCs (Table 1).

Positive blood cultures after placement were found in 25 patients: 19 with tunneled catheters and 6 with non-tunneled catheters. Of these patents, 15 received pre-procedure antibiotics, 1 was already on antibiotics, and 7 did not have antibiotics given (Table 2). There was no significant difference in whether antibiotics were given prior to placement and positive blood cultures (*p* = 0.075).

There were 153 catheter placements with no signs of infection: 78 tunneled and 75 non-tunneled. There was a statistically significant difference in WBC at the time of placement and subsequent positive blood culture for tunneled catheters (*p* = 0.01), but not for non-tunneled catheters (*p* = 0.63). Similarly, there was significant difference between thrombocytopenia at the time of catheter placement and subsequent positive blood cultures for tunneled catheters (*p* = 0.02), but not non-tunneled catheters (*p* = 0.06) There was a significant difference between hemoglobin and hematocrit at the time of catheter placement and subsequent positive blood cultures for non-tunneled catheters (*p* = 0.01 and *p* = 0.01). However, there was no significant difference between hemoglobin and hematocrit for tunneled catheters (*p* = 0.86 and *p* = 0.62) (Table 3 and Table 4).

**Figure 1:**
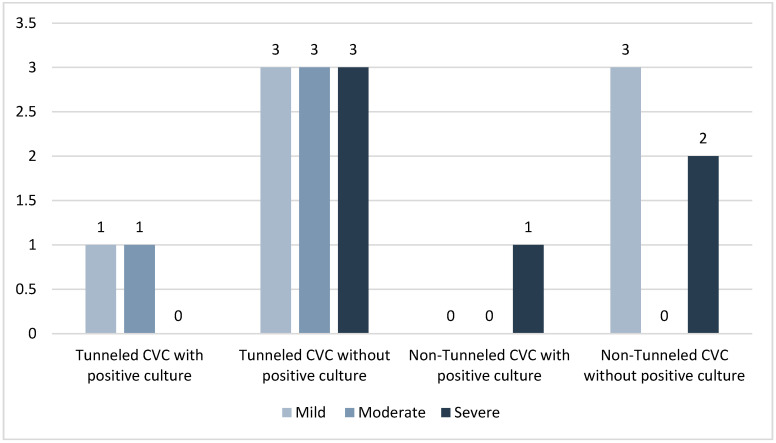
Documented bleeding events after line placement.

There were 17 incidences of bleeding after catheter placement: 11 in tunneled catheters, 6 in non-tunneled catheters. Patients with tunneled catheters that had a subsequent positive blood culture had two incidences of bleeding (18.2%) while those without infection had nine incidences (81.8%). Patients with non-tunneled catheters that had a subsequent positive blood culture had one incidence of bleeding (16.7%), while those without infection had five incidences (83.3%) (Figure 1). The patient groups of tunneled and non-tunneled catheters both had three severe bleeding events, but tunneled catheters were the only patient group to have moderate bleeding events with four total events (Figure 1).

**Figure 2:**
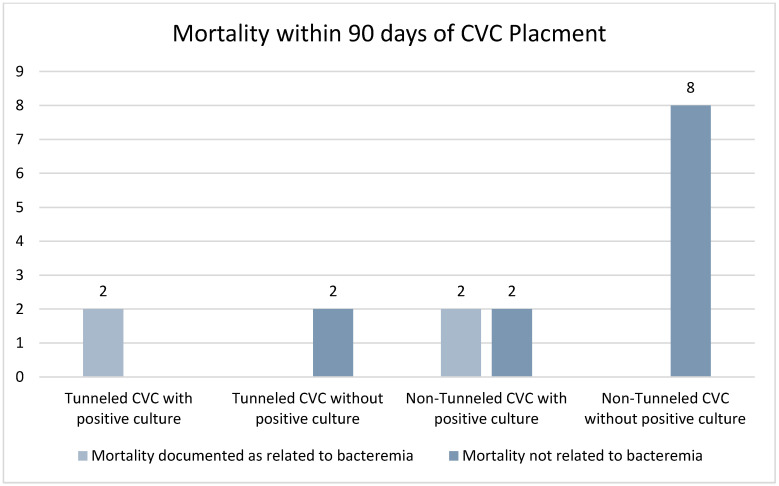
Mortality rate of patients deceased within 90 days of line placement.

There were 16 patients that died within 90 days following catheter placement. Of these patients, it was documented that four deaths were a result of bacteremia, with two having had a tunneled CVC, and two a non-tunneled CVC (Figure 2). The remainder of the deaths were documented as unrelated to the placement of the CVC or of unknown cause. No patients died within 24 h of catheter placement. Of note, 66.7% of patients with non-tunneled CVCs and subsequent positive blood cultures died within the study period, while only 10.5% of patients with tunneled CVCs and subsequent positive blood cultures died; however, this was not statistically significant (*p* = 1.0). The rate of death from all patients with bacteremia was 25%, compared with those without bacteremia at 6.58%, which was statistically significant *p* = 0.0015.

## 4. Discussion

CVC placement is a common procedure performed increasingly by radiology and interventional radiologists using imaging guidance, ultrasound and fluoroscopy [14]. There are more than 5 million CVCs placed in the US annually [15]. Multiple studies have shown that leukopenia and thrombocytopenia lead to an increased risk of infection for implantable ports [7,8]. Our study confirmed similar findings for tunneled CVC; leukopenia and thrombocytopenia led to increased risk for bacteremia after tunneled CVC placement.

CVCs can be either tunneled for long-term intravenous access or non-tunneled for short-term access. Non-tunneled catheters are typically used for several days to 3 weeks. These catheters are placed either when temporary access is needed, or if a patient is deemed not a candidate for a permanent (tunneled) CVC due to abnormal laboratory values [16]. These catheters are placed directly into the vein.

Permanent CVCs are tunneled through the soft tissues of the chest before entering the vein. These have a cuff that sits in the soft tissue and incites an inflammatory response, causing a scar helping to fix the catheter in place and preventing bacteremia [17]. Tunneled CVCs differ from implantable ports, in that part of the access remains external to the patient at all times.

Leukopenia is a marker of overall immunosuppression. It has been shown to be a risk factor for infection of implantable ports [7,8,18,19]. Our study found that leukopenia was also a risk factor for subsequent bacteremia in patient’s undergoing tunneled CVC placements. Leukopenia was not found to be a risk factor for bacteremia in non-tunneled CVC placements. This is not surprising, as leukocytosis is an indication to place a temporary line over a tunneled line [16].

Thrombocytopenia at the time of tunneled CVC placement was associated with a significantly increased risk of subsequent positive blood culture. Platelets are known to serve not only in the coagulation cascade, but to have an immune function. Platelets circulating in the blood stream are recruited to sites of infection where they bind to pathogens, preventing dissemination and aiding the immune response [20]. Studies have found that thrombocytopenia is a risk factor for infection [21,22]. Additionally, studies have found that thrombocytopenia at time of CVC placement is a risk factor for bleeding and hematoma formation [23]. Our study had 17 patients with documented bleeding complications, 11 of which were in tunneled CVCs. This could serve as a nidus leading to infection.

The current Society of Interventional Radiology guidelines for the use of antibiotics recommend that prophylactic antibiotics, 1–2 g cefazolin, be given prior to tunneled CVC placement. The guidelines, however, do not recommend that antibiotics be given for non-tunneled CVC placement, except in immunocompromised patients [24]. Our study did not find a significant different between bacteremia in patients who had received antibiotics and those that did not. As our clinical practice follows these guidelines, this may be more reflective of the small outlying sample size; only five tunneled CVC patients did not have pre-procedure antibiotics, and only one non-tunneled CVC received pre-procedure antibiotics.

Within our study, 16 patients died within 90 days of catheter placement. Eight of these patients had non-tunneled CVC. This likely reflects the underlying tenuous clinical status which may have been related to needing a temporary CVC. Four of the deaths, two tunneled CVCs and two non-tunneled CVCs, were related to bacteremia and sepsis. This highlights the severity of catheter-related bloodstream infections.

Our study had several limitations. First, this was a retrospective single-institution study. Bacteremia was identified by positive blood cultures after CVC placement. Patients with fever of unknown origin were excluded, as this can be seen commonly in the setting of post-bone marrow transplants. The sample size was small, and the indications for catheter placement were diverse, including dialysis, plasmapheresis, stem cell collection, and bone marrow transplant, among others. Many patients had complex hospital courses and medical co-morbidities. Our institutional guidelines require laboratory values within 30 days of a tunneled line placement, but not the day of placement. While many were within 24 h of placement, this could have led to bias in our data. Additionally, during the study period, our institution protocol for the antibiotic administration of tunneled catheters changed after a review of our data and complications. We stopped routinely giving pre-procedure antibiotics.

## 5. Conclusions

Leukopenia and thrombocytopenia at the time of placement are risk factors for subsequent CLABSI in patients undergoing placement of a tunneled large-bore CVC. These values should lead to the consideration of using a non-tunneled CVC, or the delayed placement of these catheters until counts recover, when clinically possible.

## Figures and Tables

**Table 1:** Demographic information.

	Positive Blood Cultures *n* = 25	No Positive Blood Culture *n* = 152
**Age**	23–75 mean 53.3	17–85 mean 53.0
**Male**	14 (56.0%)	93 (61.2%)
**Female**	11 (44.0%)	59 (38.8%)
**Tunneled CVCs**	19 (76.0%)	77 (50.7%)
**Non-tunneled CVCs**	6 (24.0%)	75 (49.3%)
**Catheter French**	12–14.5	12–16
**Underlying Diagnosis**	Hematologic (*n* = 15): - Leukemia (*n* = 7) - Lymphoma (*n* = 4) - Other (*n* = 4) Renal (*n* = 10): - Acute kidney injury (*n* = 4) - CKD/ESRD (*n* = 6)	Hematologic (*n* = 48) - Lymphoma (*n* = 2) - Leukemia (*n* = 31) - Stem cell donor (*n* = 7) - Other (*n* = 8) Renal (*n* = 77): - Acute kidney injury (*n* = 31) - CKD/ESRD (*n* = 46) Neuro (*n* = 14) - Encephalitis (*n* = 3) - Inflammatory polyneuropathy (*n* = 4) - Myositis (*n* = 3) - Other inflammatory disorder (*n* = 4) Other (*n* = 13): - Solid organ transplant rejection (*n* = 10) - Other (*n* = 3)
**Reason for CVC**	- Bone marrow transplant (*n* = 13) - Stem cell collection (*n* = 2) - Hemodialysis (*n* = 10)	- Plasmapheresis (*n* = 25) - Bone marrow transplant (*n* = 14) - Stem cell collection (*n* = 31) - Hemodialysis (*n* = 78) - Other: (*n* = 4)

**Table 2:** Antibiotics at time of CVC placement.

	Pre-Procedural Antibiotics Given	Already Receiving Antibiotics	No Antibiotics Given
**Positive blood cultures and tunneled CVC** ***n*** **= 19**	15 (78.9%)	3 (15.8%)	1 (5.3%)
**No positive blood culture and tunneled CVC** ***n*** **= 77**	62 (80.5%)	11 (14.3%)	4 (5.2%)
**Positive blood cultures and non-tunneled CVC** ***n*** **= 6**	0 (0%)	0 (0%)	6 (100.0%)
**No positive blood cultures and non-tunneled CVC** ***n*** **= 75**	1 (1.3%)	0 (0%)	74 (98.7%)

**Table 3:** Biomarkers statistics for tunneled CVCs.

	Positive Blood Cultures *n* = 19	No Positive Blood Culture *n* = 77	*p* Value
**WBC**			
Mean (sd)	4.53 ± 3.08	9.55 ± 8.22	*p* = 0.01
Median	4.05	7.86	
Range	0.51–12.87	2.25–60.11	
**Hemoglobin**			
Mean (sd)	10.50 ± 2.17	10.61 ± 2.58	*p* = 0.86
Median	10.20	10.3	
Range	22.10–44.3	6.5–19.0	
**Hematocrit**			
Mean (sd)	31.85 ± 6.13	32.81 ± 7.98	*p* = 0.62
Median	31.70	31.8	
Range	22.10–44.30	18.5–58.9	
**Platelets**			
Mean (sd)	150 ± 84.93	209.54 ± 100.93	*p* = 0.02
Median	181	197	
Range	12–270	32–541	

**Table 4:** Biomarkers statistics for non-tunneled CVCs.

	Patients with Positive Blood Cultures *n* = 6	Patients without Positive Blood Cultures *n* = 75	*p* Value
**WBC**			
Mean (sd)	9.74 ± 10.27	23.10 ± 68.13	*p* = 0.63
Median	7.61	11.31	
Range	1.55–29.97	2.44–593.52	
**Hemoglobin**			
Mean (sd)	8.77 ± 1.6	11.22 ± 2.32	*p* = 0.01
Median	8.35	11.4	
Range	7.2–11.7	3.94–15.9	
**Hematocrit**			
Mean (sd)	27.33 ± 5.86	34.61 ± 6.76	*p* = 0.01
Median	25.75	35.1	
Range	22.2–38.7	19.3–35.1	
**Platelets**			
Mean (sd)	125.82 ± 75.05	212.85 ± 111.34	*p* = 0.06
Median	112.5	197	
Range	26–247	6–487	

## Data Availability

The data presented in this study are available on request from the corresponding author. The data are not publicly available due to patient privacy.
